# Nanovaccines against Animal Pathogens: The Latest Findings

**DOI:** 10.3390/vaccines9090988

**Published:** 2021-09-04

**Authors:** Carmen Teresa Celis-Giraldo, Julio López-Abán, Antonio Muro, Manuel Alfonso Patarroyo, Raúl Manzano-Román

**Affiliations:** 1 Molecular Biology and Immunology Department, Fundación Instituto de Inmunología de Colombia (FIDIC), Bogotá 111321, Colombia; ccelis@udca.edu.co; 2 Animal Science Faculty, Universidad de Ciencias Aplicadas y Ambientales (U.D.C.A), Bogotá 111166, Colombia; 3 Infectious and Tropical Diseases Research Group (e-INTRO), Institute of Biomedical Research of Salamanca-Research Center for Tropical Diseases at the University of Salamanca (IBSAL-CIETUS), Faculty of Pharmacy, University of Salamanca, 37007 Salamanca, Spain; jlaban@usal.es (J.L.-A.); ama@usal.es (A.M.); 4 Microbiology Department, Faculty of Medicine, Universidad Nacional de Colombia, Bogotá 111321, Colombia; 5 Health Sciences Division, Main Campus, Universidad Santo Tomás, Bogotá 110231, Colombia

**Keywords:** nanoparticle, nanovaccine, pathogen, immune response, nanoplatform

## Abstract

Nowadays, safe and efficacious vaccines represent powerful and cost-effective tools for global health and economic growth. In the veterinary field, these are undoubtedly key tools for improving productivity and fighting zoonoses. However, cases of persistent infections, rapidly evolving pathogens having high variability or emerging/re-emerging pathogens for which no effective vaccines have been developed point out the continuing need for new vaccine alternatives to control outbreaks. Most licensed vaccines have been successfully used for many years now; however, they have intrinsic limitations, such as variable efficacy, adverse effects, and some shortcomings. More effective adjuvants and novel delivery systems may foster real vaccine effectiveness and timely implementation. Emerging vaccine technologies involving nanoparticles such as self-assembling proteins, virus-like particles, liposomes, virosomes, and polymeric nanoparticles offer novel, safe, and high-potential approaches to address many vaccine development-related challenges. Nanotechnology is accelerating the evolution of vaccines because nanomaterials having encapsulation ability and very advantageous properties due to their size and surface area serve as effective vehicles for antigen delivery and immunostimulatory agents. This review discusses the requirements for an effective, broad-coverage-elicited immune response, the main nanoplatforms for producing it, and the latest nanovaccine applications for fighting animal pathogens.

## 1. Introduction

The vaccine impact today is broad and far-reaching, being an essential public health tool with significant benefits for society, thus highly influencing the worldwide economy and even impacting biodefence strategies [1]. They can prevent 6 out of the 10 leading causes of death in resource-poor countries and are second only to clean water in saving lives. The WHO estimates that they save between 2 and 3 million lives each year, which means they may have prevented more than 23 million deaths from 2011 to 2020 [2,3,4]. Vaccines have figured in programs leading to the eradication of diseases such as smallpox and rinderpest. Other diseases such as polio will be eradicated in a few years’ time and vaccines are also undoubtedly key tools for improving productivity and controlling and/or eradicating many trans-boundary diseases; therefore, the World Organisation for Animal Health (OIE) is actually promoting vaccine banks which, in turn, may help to reduce poverty [5]. However, there are still cases of persistent infections, rapidly evolving pathogens having high variability or emerging pathogens (mainly from wildlife reservoirs such as African swine fever virus, with almost 7000 cases detected so far this year in wild boar) or re-emerging pathogens for which no effective vaccines have been developed, i.e., Ebola virus, Zika virus (ZIKV), Chikungunya virus (CHIKV), and Dengue virus (DENV). Although anti-SARS-CoV-2 vaccines have been shown to have very good initial protective efficacy, antibody titres decrease a few months after vaccination and the emergence of new variants has raised concerns about the vaccination strategy used [6,7,8,9]. These are impressive human diseases, although their broad tropism, host evolution-related adaptation via single mutations (and the possibility of new mutations altering tropism), many unknown host restriction mechanisms, and the lack of restricted human receptors suggest (at least for some of them) important a priori potential for infecting animal cells and developing disease. This is exemplified by zoonotic coronaviruses and the latest SARS-CoV-2 virus showing receptor orthologs widely distributed in various domestic and wild mammals in which COVID-19 appears. Furthermore, it is clear that viral persistence may be facilitated by a number of macro-ecological characteristics, in turn contributing to variation and a proclivity for specific hosts [10,11,12,13,14,15]. The potential transmission of pathogens currently affecting humans to animal populations has highlighted the need to adopt a One Health approach regarding global health interventions. Recent outbreaks of pertussis and measles in countries having a history of well-established vaccination campaigns and low efficacy or transient protective effect of some vaccines already on the market (e.g., BCG and influenza vaccines) are evidence of re-emerging pathogens. Therapeutic options for many animal and human diseases are either lacking or problematic (e.g., due to the development of resistance) or have limited efficacy. This highlights the continuing need for novel disease management alternatives, including vaccines for preventing epidemics and antibiotic resistance induction [16,17].

Most licensed vaccines are traditional inactivated or live attenuated vaccines; viral vector vaccines and subunit-based vaccines have proven to be very useful because of their intrinsic ability to act as adjuvants, infect cells, or activate innate immune responses. These are thus essential tools for controlling seasonal influenza and many important veterinary and zoonotic diseases such as foot-and-mouth disease (FMD) or rabies, in turn positively impacting animal productivity, food security, and several human diseases [18]. However, no efficacious vaccines are available for most highly contagious and epidemic animal diseases [19]. This is mainly due to the intrinsic limitations of traditional vaccinology approaches and other limitations, such as adverse effects (e.g., the licensed rotavirus vaccine), the low avidity of vaccine-induced antibodies (Abs) against respiratory syncytial virus (RSV), or the partial protection they induce against a large and significant amount of pathogens such as Dengue virus or the *Plasmodium* parasites causing malaria in which induced protection is less than 50% (Table 1; [20,21,22]). Modern subunit-based vaccines promote rapid clearance, enzyme degradation, poor solubility, immunogenicity, and low uptake by antigen-presenting cells. Another constraint is that most pathogens first infect mucosal surfaces and few mucosal vaccines have been licensed since immunity induced via this route requires a special delivery system enabling antigen bioavailability and capture by antigen presenting cells (APCs) [23]. All these pose a number of challenges broadly shared amongst veterinary vaccines [24,25].

Nanotechnology nowadays allows the development of vaccines based on nanomaterials having encapsulation ability and very interesting properties due to their size and surface area, enabling them to serve as effective vehicles for antigen delivery and immunostimulatory agents. The hepatitis B virus vaccine was the first recombinant vaccine approved for use in humans; it was formulated with a surface antigen that was initially prepared from infected donor plasma. Its production in 1985 using recombinant technologies and inclusion in virus-like particles (VLPs) that are 1000 times more immunogenic than monomer Ag constituted the first commercialised human nanovaccine. Nevertheless, nanotechnology began earlier (as early as 1974) with the manipulation of nanoscale materials (nanobiotechnology today describes the use of nanotechnology in the life sciences). The term nanoscale describes materials that have one or more of their three dimensions measuring between 1 and 100 nm—viruses between 10 and 100 nm. Other recently developed nanobiomaterials (typically 5–20 nm) are being designed to have structural similarity to different receptors, ligands, DNA, and proteins. The size of the nanoparticle (NP) decisively influences nanovaccine biodistribution and interaction with immune system cells; particles smaller than 100 nm are very efficiently taken up by their targets, the dendritic cells. The nanomaterials themselves act as effective adjuvants capable of activating humoral and cellular responses. The circulation time of biomaterials can also be improved, their bioavailability increased, or biological material protected from degradation. Controlling antigen and adjuvant size, shape, surface charge, flexibility, hydrophobicity, and charge density in nanovaccines is the key to delivering nanoparticles to the lymphatic vessels and optimising a strong immune response [26]. A vast number of publications has focused on properties which have been significantly used for fighting animal diseases during the last two years. Our aim was to present the molecular basis and requirements for achieving desired protection using vaccines and emphasise modern nanoplatform and nano-approaches’ potential, along with the latest practical outcomes from a One Health vaccinology perspective, since most human infectious diseases have an animal origin [27]. Information regarding human vaccines has also thus been mentioned to describe the wide range of potential platforms and approaches available for veterinary vaccine development.

## 2. An Effective Broad-Coverage Immune Response

Humans and animals must deal with a limitless number of microorganisms in their environments involving them in situations wherein the pathogen–host interaction will determine the type of outlook for each one [23,28]. It is clear that a series of events occurs which leads to infection, ranging from microorganism entry, subsequent colonisation and tissue invasion, tissue damage, and its evasion of a host’s immune system [29,30]. The immune response to microorganisms can be condensed into four common aspects: host development of specialised defence mechanisms via innate and adaptative immunity, developing pathological alterations, persistent infections and tissue lesions (depending on host susceptibility and pathogen virulence), microorganisms being able to evade an immune response or resist host defence mechanisms, and immune system variations (inherited or acquired) promoting or protecting infections [31,32,33]. This section covers generalities regarding host immunological response mechanisms for dealing with various microorganisms (bacteria, viruses, and parasites), along with evasion mechanisms, including aspects associated with host and pathogen genetic variability and the nanovaccine platform’s immunological scope.

### 2.1. Considerations Regarding Host Immune Response to Various Pathogens

The evolution and clinical manifestation of infection varies in individuals affected by transmissible agents; they could lead to high morbidity and mortality, reduce fertility, and productive efficiency, and cause largescale economic loses for production systems, thereby making infection eradication and/or control a challenge [23]. Most pathogenic agents cause acute infection which can be efficiently controlled by a host’s immune system; intracellular pathogens can provoke persistent infections, sometimes lasting for life [33].

The innate immune system becomes activated during early stages of the infection by recognising alarm signals in two ways: exogenous (microorganism-derived, via pathogen-associated molecular patterns (PAMPs) and endogenous (dead/dying cell-derived, via damage-associated molecular patterns (DAMPs)). Several pattern recognition receptors (PRRs) are expressed in host cells, i.e., Toll-like receptors (TLR) enabling recognition of PAMPs and DAMPs, thereby activating an immune response via complement proteins, as well as phagocytic (monocytes, macrophages, and neutrophils) and natural killer (NK) cells [23,33]. Such recognition is rapid, non-specific, and leads to responses such as phagocytosis, cell locomotion, parasite, and/or cell elimination and cytokine production. Protection against reinfection has been reported in plants and invertebrates, suggesting adaptation of host innate mechanisms which can provide certain adaptative characteristics called “trained immunity” in humans and rodents. This mechanism responds to a second infection; however, it has been associated with complications in some cases, such as immune-mediated pathologies and chronic inflammatory diseases [34]. Few veterinary studies have dealt with it, despite being of great interest due to providing immunological alternatives which can promote resistance to disease and reduce antibiotic use [35].

Cell-mediated immunity neutralises a pathogen or antigen, thereby promoting the development of immunological memory; this is triggered by activated dendritic cells migration to the lymphoid organs (spleen and lymphoid nodules) where they present class I antigens to CD8^+^ cytotoxic T-lymphocyte receptors, the final result being to eliminate infected or cancer cells by apoptosis [33]. Dendritic cells can also present their antigens via major histocompatibility complex class-II (MHC-II) to CD4^+^ T-helper cells causing their maturation due to the effect of the cytokines produced. Interferon gamma (INF-ϒ), tumour necrosis factor alpha (TNF-α) and interleukin-1 (IL-1) promote T-helper 1 (Th1) cell development, increasing phagocytic and cytotoxic activity. Th2 cells secret cytokines such as IL-4, IL-5, IL-6, IL-9, IL-10, and IL-13, promoting B-cell proliferation during an Ab–mediated immune response [18,36,37]. Human T-helper response has been studied in greater detail and is not just restricted to Th1 or Th2 cells but also to other subgroups, such as regulatory T-cells (Tregs), Th9, Th17, and Th22 [38].

Regulatory T-cells (Tregs) are the main regulators of immune responses against self, pathogenic, and commensal antigens in the periphery and have been widely studied in both humans and mice. It has been shown that CD4^+^Foxp3^+^ cells exert a suppressive activity in mice. Information regarding Tregs in other animal species is scarce, mainly due to the limited availability of monoclonal antibodies against their specific surface antigens; however, a study of CD4^+^CD25^high^ T-cells, isolated ex vivo from healthy dogs, has shown that these cells have a regulatory phenotype [39]. These cells have been studied regarding infection in various animal species by analysing their CD25 (IL-2R) and Foxp3 expression, as well as IL-10 production [40,41,42,43]. Despite having CD4^+^CD25^high^Foxp3^+^ cells, a greater percentage of ϒδ T cells (15–60%) in cattle’s peripheral blood has been described, mainly being classified as a regulatory population able to secrete IL-10 and proliferate upon IL-10, tumour growth factor-beta (TGF-β) stimuli and APC interaction [44].

**Figure 1:**
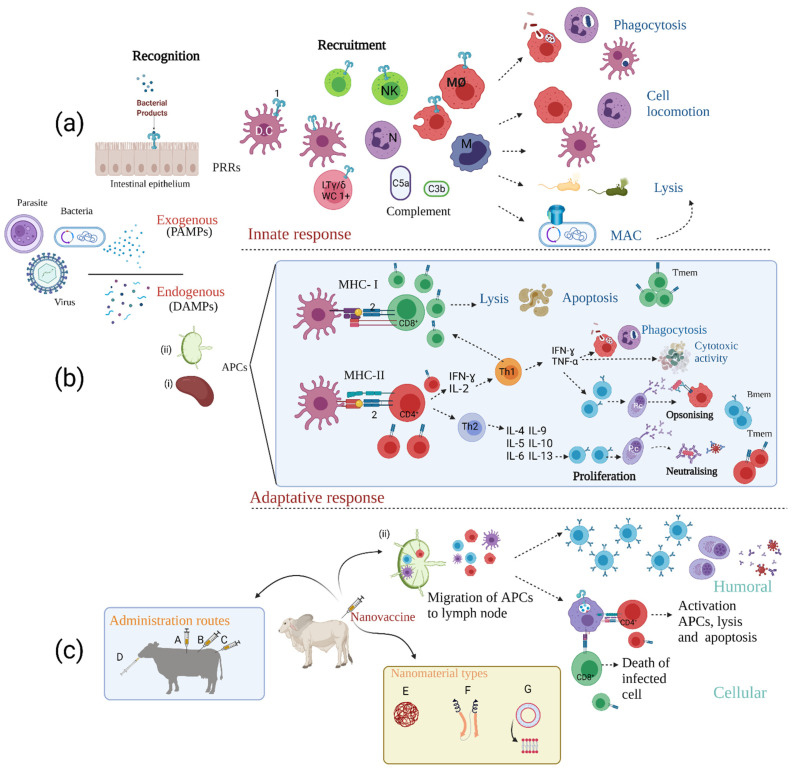
General representation of the immune response to pathogens and the effect of nanovaccines on the immune system. Recognition of exogenous pathogens (e.g., bacteria (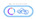), viruses (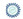), and parasites (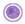) is via their pathogen-associated molecular patterns (PAMPs). Endogenous particle recognition from dead or apoptotic cells is by damage-associated molecular patterns (DAMPs), thus activating the arms of the immune response: (**a**) innate immunity. After PAMPs interaction with host pattern recognition receptors (PRRs) (i.e., Toll-like receptors (1)), the action of complement proteins (i.e., C5a and C3b) and the final effect of recruited immune cells such as dendritic cells (DC), macrophages (MØ) and LTϒ/δ (specially in swine and ruminants (LTϒ/δ-WC1+)) starts; natural killer cells (NK), monocytes (M), and neutrophils (N) eliminate infectious agents, mainly by phagocytosis, cell locomotion, the formation of the membrane attack complex (MAC) and lysis. (**b**) Adaptive immunity. Antigen presenting cells (APCs) migrate to secondary lymphoid organs such as the spleen (i) and lymph nodes (ii) with subsequent antigen presentation (via MHC-I) to the TCR (2) of CD8^+^ cytotoxic T-lymphocytes (triggering cell-mediated apoptosis) and via MHC-II presentation to CD4^+^ T cells which leads to their maturation. IFN-ϒ and IL-2 secretion then promotes commitment to a Th1 profile increasing phagocytic and cytotoxic activity and promoting opsonising Ab production by B-cells. Th2 cell responses promote B-cell proliferation, plasma cell (Pc) maturation, and consequent production of neutralising Abs (NAbs). After infection control, most immune cells die by apoptosis and a small percentage differentiate to memory T (Tmem) and memory B (Bmem) cells. (**c**) Immunological response to nanovaccines. These vaccines have several advantages aimed at eliciting long-term balanced protective immune responses, including (i) different administration routes (intramuscular [A], subcutaneous [B], intravenous [C], oral [D]) (ii) different nanomaterial types and properties (i.e., polymer nanoparticles [E], protein-based nanoparticles [F] and liposomes [G]) which prolong interaction time with immune system cells, protect the antigen and potentially serve as adjuvants and immunomodulators and (iii) stimulate APC migration to the secondary lymphoid organs and increase lymphocyte maturation. Designed by: Sofía Chaves-Vargas and Sofía Riaño-Riaño, Animal Science Faculty, U.D.C.A. Created with BioRender.com, accessed on 26 July 2021.

Spleen and lymphatic nodules’ naïve B-cells are stimulated and bind to soluble antigens via B-cell receptors (BCR) in humoral immunity; this can be strongly influenced by nanovaccine action, leading to germinal centre (GC) proliferation. Only epitope-specific B-cells are selected for clonal expansion; once activated, they become differentiated into plasma cells and secret soluble Abs against a target antigen, neutralising extracellular pathogens. When infection is controlled, a small percentage of plasma cells remain as memory cells which can respond more rapidly and strongly during reinfection [37]. Figure 1a,b illustrates the forgoing information.

The ability to remain in a host is characteristic of intracellular microorganisms; such persistence can be asymptomatic, putting a host at risk when the dormancy phase becomes reactivated [45,46]. There are two types of dormancy depending on the type of pathogen [33]; the first encompasses pathogens detected by the adaptative immune system which remain in a state of dormancy and which do not become completely eliminated from a host (e.g., *M. tuberculosis* [45] and *S. enterica* [47]. The second type involves opportunistic agents found in the flora which do not cause an adaptative immune response in healthy individuals but are able to cause active infection in immunocompromised individuals (e.g., *Neisseria* [48]). Just as there is a great diversity of pathogens, there is a broad variety of evasion mechanisms. Some would include evasion of immune recognition systems through surface modulation, the secretion of immunomodulator agents, antigen variation and concealment in tissue or target cells. The other group bases its mechanisms on modulating and supressing an immune response by avoiding phagocytosis, innate immunity receptor action, the complement system, cytokines, chemokines, inhibiting apoptosis, pathogen resistance to effector mechanisms, and the induction of immunosuppression [33]. An in-depth review of such mechanisms is beyond the scope of this document.

### 2.2. Genetic Variability and Immune Response

The group of MHC-related genes encodes an extensive repertoire of surface proteins playing a crucial role in recognising mammalian antigens; in spite of large differences between species, their variability has been associated with susceptibility to or protection against infectious diseases and even cancer [49,50]. Most research regarding class II molecules has focused on the DRB1 locus (DRB3 in cattle) as being the most variable and having the greatest cell-related expression; on the contrary, DRA is considered practically invariant [50,51,52,53]. Manczinger et al. (2019) proposed that human DRB1 MHC-II allele response could be categorised into two groups; specialists (alleles able to bind to a few epitopes) and generalists (providing protection against a broad range of pathogens). The mutual selective pressure exerted between pathogens and host maintains high allele histocompatibility variability in the population and induces genetic escape variants to become fixed in pathogens [54].

Regarding veterinary studies, a study has explored the MHC in different species. Allele frequency and diversity in pigs has been analysed in wild and production species which are of interest, due to their biological characteristics, their use in biomedical studies, transplants, regenerative medicine, and vaccine design [53,55,56]. Hammer et al. (2021), for example, used low-resolution (Lr) SLA haplotyping for characterising European SLA-I (SLA-1, SLA-2, and SLA-3) and SLA-II genes (DRB1, DQB1, DQA) in 549 pigs representing nine commercial lines from various production systems; they identified 50 class I haplotypes and 37 class II ones. They have also carried out vaccine correlation studies regarding economically important viral diseases in pig species such as porcine reproductive and respiratory syndrome (PRRS) virus, classical swine fever virus (SFV), foot-and-mouth disease virus (FMDV) and swine influenza A virus (SIAV); they found immunodominance regarding haplotype-dependent T-antigen-specific response. The alleles found in SLA-I, Lr-04.0, and Lr-32.0 were the most abundant in European pigs, whilst Lr-0.15b and Lr-0.12 were the most abundant in SLA-II [55].

DRB3 has been characterised in bovine species in various breeds and populations worldwide [57,58,59]; Mandefro et al. (2021) showed a difference regarding BoLA-DRB3 genetic variability between African and Asiatic breeds. A study of DRB3 locus diversity by Peters et al., (2018) analysed Asian populations and compared African and American populations; 15 haplotypes were reported from the 174 cattle analysed, the Brangus (10), Sokoto Gudali (10), and Dajal (9) breeds having the greatest number of haplotypes whilst only four haplotypes were found in the Holstein and Sahiwal group. They attributed 94.01% of genetic variation to differences within populations and 5.99% to differences between populations [58]. A study by Daous et al. (2021) associated bovine leukosis virus (BLV) proviral load (PVL) with BoLA-DRB3 polymorphisms, finding that individuals typed BoLA-DRB3.2*3, *7, *8, *11, *22, *24, and *28 had low PVLs, whilst BoLA-DRB3.2*10 individuals were associated with high PVLs. A similar pattern was observed for heterozygotes, finding low PVLs to be associated with genotypes *3/*28, *7/*8, *8/*11, *10/*11, and *11/*16, and high PVLs in genotypes *1/*41, *10/*16, *10/*41, *16/*27, and *22/*27 [60].

Selecting functionally conserved regions is another aspect to be considered when developing vaccines (especially peptide-based ones). The *Plasmodium falciparum* parasite uses conserved protein regions to bind to human erythrocytes and invade them; unfortunately, it has been found that these functionally important regions are usually poorly immunogenic [61]. Approaches have thus been used which enable target cell binding sequences’ presentation in MHC II molecules to become optimised for counteracting this problem [62,63].

Mutation and recombination are clearly relevant for pathogen evolution; such changes can provide a host with immune system resistance and against drugs, or more seriously, they can lead to outbreaks [64]. What is happening with the SARS-CoV-2 pandemic provides a clear example, where mutations and genetic variability have increased transmissibility and are affecting response to vaccines [65]. Such a situation has forced new alternatives to be developed, thereby promoting recent developments in nanotechnology [66].

Advances in the area of omic sciences have facilitated a large amount of information to be obtained on pathogens, further clarifying immunological and pathological mechanisms and promoting the development of vaccines and therapeutic strategies [32,67,68]. The use of bioinformatics tools has facilitated the development of T-epitope predictors, i.e., PigMatrix (class I and class II) and EpiCC in porcine species, which make up the new in vitro conjugate vaccine expression (iVAX) manufacturing platform (i.e., cell-free synthetic biology) used as a tool for human vaccine development [69]. Cattle-related tools/servers have been developed for predicting peptide binding to class I BoLA (NetBoLApan) [70] and BoLA-DRB3 class II molecules (NetBoLAIIpan); the latter was validated by integrating immunopeptidomics data and the predictor has been used for identifying peptide candidates for an anti-*Riphicephalus microplus* vaccine (a tick having a great impact on production systems worldwide) [71]. Producing predictors for different species will enable a wide range of epitopes that are well presented in the most frequently occurring histocompatibility alleles in populations to be included. The same type of design should provide broad coverage and protection for the selected candidates in a population of interest.

### 2.3. Nanovaccine Considerations Regarding the Immune Response

Vaccine design seeks to ensure that host adaptative immunity be long-lasting and highly specific, thereby enabling it to act against possible reinfections [36]. The main strengths derived from using nanovaccines has been dealt with in the pertinent literature [72,73,74]. The NP-based vaccine approach (nanovaccines) is very promising because of the great advantages offered by being properly captured by the immune system cells. They can induce both rapid and durable humoral and cellular immunity up-regulating genes and enhancing antigenic processing, they can be delivered through multiple routes, maintain long-lived antigen stability and functionality, and enable antigen and adjuvant specific transport to antigen-presenting cells (mainly dendritic cells able to control immune tolerance and immunity). These cells will process antigens and carry out cross-presentation to T-cells for activation enabling a cellular immune response. All this takes place in a five-step “cascade” involving antigen functionalisation, drainage to lymph nodes, internalisation by presenting cells, presenting cell maturation and MHC-I-peptide immunocomplex presentation to T-cells [75]. The latter may be equally problematic since cell infection by pathogens and classical endogenous processing itself down-regulates MHC expression, potentially altering the immune responses (80). Intrinsically shaped nanoparticles facilitate most of these cross-presentation steps in the lymph nodes by changing the property of dendritic cells to present exogenous antigenic molecules to CD8^+^ T-cells. Nanoparticle-induced endosomal osmotic swelling and rupture exposing the antigen and membrane fusion mechanisms facilitate cytosolic exportation and such unconventional antigen presentation leads to cellular immune responses [76,77] (see Figure 1c). The following must thus be taken into account when constructing a nanovaccine release system: the type of antigen, adjuvant, and release system [37,78].

Antigenicity has classically been considered to depend on three characteristics: structural complexity, foreignness (recognised as non-self by the body), and high molecular weight [79]. Proteins thus make the best antigens, followed by the polysaccharides.

Regardless of the chosen methodology (recombinant proteins, RNA vaccines, DNA vaccines, VLPs, etc.), most vaccines ultimately seek to deliver an antigen directly in protein form, or by inducing a host to produce it in its own cells (RNA and DNA vaccines) [80]. Polysaccharide-based vaccines have sometimes been seen to confer a good protective immune response, particularly regarding the prevention of *Streptococcus pneumoniae*-related bacterial pneumonia and meningitis [81].

Two properties are required for an adjuvant: trapping an antigen at the immunisation site (so that immune system cells recognise it and carry it to adjacent lymphatic ganglions) and having an immunopotentiator effect (mainly by activating an innate immune response). The main interest in developing adjuvants such as the carbomer-based nano-emulsions (included in several veterinary vaccines) is to stimulate potent CD8^+^ and CD4^+^ T-cell protective responses not just by enhancing cross-presentation by the accumulation of processed antigen in DCs and promoting its trafficking to less acidic early endosomal compartments in these cells, but also by inducing a number of key metabolic switches [82]. A detailed review of adjuvants is beyond the scope of this review; the following can be consulted if such area is of interest [83]. In spite of great laboratory-related advances, relatively few adjuvants have reached the licencing phase; the most important ones have been aluminium salts, AS04 (monophosphoryl lipid A (MPL) with aluminium hydroxide), AS03 (oil-in-water emulsion consisting of squalene, alpha-tocopherol and Tween 80), MF59 (oil-in-water emulsion consisting of squalene, polysorbate 80, sorbitan trioleate, and the virosomes [84].

Vaccines formulated with alum as adjuvant induce a Th2 profile response (mainly IL-4 secretion) whilst nanovaccines can stimulate proinflammatory cytokines such as IL-1 and IL-18 and induce a cellular immune response, manganese (Mn) nanoparticle use being a good example [85]. A study by Wang et al. (2021) has described the development of a nanovaccine consisting of a SARS-CoV-2 spike (S) protein receptor-binding domain (RBD) and manganese as nanoadjuvant (MnARK); they found a strong cellular immune response, even at low doses, accompanied by efficient cellular internalisation [86]. Something similar is sought with licenced adjuvants, i.e., adjuvant system AS04 combining alum with monophosphoryl lipid A (MPL) [87]. MPL is a modified, significantly less toxic version of lipopolysaccharide (LPS) whilst still remaining a TLR4 agonist [88]. Both Th1 and Th2 responses can be elicited by including MPL with aluminium hydroxide [87].

A third characteristic concerns release systems involving different types of nanoparticles (detailed later on in the document). Zhang et al. (2020) constructed a vaccine based on single-wall carbon nanotubes interacting with antigen-presenting cells’ mannose receptor, combined with rhabdovirus mannosylated antigen in fish; such a vaccine had greater reuptake by macrophages and tissue associated with an immune response 6 h after immersion, achieving up to 63.5% survival rates compared to the control group [89].

Other aspects such as increased antigen stability and reduced degradation have led to nanovaccines triggering sustained antigen release (i.e., creating an antigen reservoir), meaning that the time of remaining at the application site lasts longer, thereby facilitating immune cell action [73]. Concerning particle size, a diameter of up to 10 µm enables them to enter cells by endocytosis (mainly observed in macrophages) [26,74].

Nanomaterial interaction is very important for triggering a response regarding innate immunity [36]. Depending on immunisation route, a nanovaccine comes into contact with neutrophils, macrophages, DC, and natural killer (NK) cells; its components will be endocyted, degraded, and presented following recognition [37]. Nanomaterials can exert adjuvant activity and activate an immune response by glycolic acid action (i.e., a PGLA component) or by cationic liposomes, causing cytokines and secreted chemokines to contribute towards dendritic cell maturation and activation [37,66].

An adaptative immune response enables a host to recognise pathogens, create long-lasting pathogen-specific memory able to destroy a pathogen every time it is confronted, being positively influenced by the use of nanovaccines and nanoadjuvants [74,90]. For example, if antigen structure undergoes changes due to cleavage, aggregation, or folding, memory cells will not be able to provide the required protection; poly(lactide-co-glycolide) acid (PLGA)-based vaccines can provide prolonged antigen bioavailability without changes in the blood due to sustained release, thereby promoting B-cell proliferation and an increase in memory cells [37].

## 3. Nanoplatforms: Modern Approaches for Producing Real Anti-Pathogen Vaccines

Classic vaccine platforms suffer from manufacturing difficulties such as an individual antigen of interest’s inherent biophysical traits. The recent COVID-19 pandemic has highligted the need to speed up the development of vaccines for emerging infectious disease outbreaks. It has accelerated the development of next-generation vaccine platforms driven mainly by their potential use in cancer therapies and based on sequence information alone, which makes them highly flexible with the likelihood of co-displaying multiple distinct immunogens and inducing broad protection [91,92]. Vaccine evolution has led to modern platform technologies meeting these criteria which are more likely to counteract the risks associated with emerging infectious diseases, thus achieving economies of scale. Nanoplatform infrastructures provide improved efficacy in relation to conventional vaccines and marketable tools to fight infectious diseases involving negligible financial risk. Although not the panacea, the future of vaccine research and development may pass through nanoplatform-based approaches for many pathogens [75,93,94]. NPs have different roles in vaccinology mainly as adjuvants, carriers, and platforms. Many nanoscale-based adjuvants and delivery vehicles for vaccines are able to elicit desirable immune responses (Table 2; [95]).

Sophisticated nanovaccine-based strategies are now available to support innate and specific T-cell-mediated immunity during each of these stages [110]. For example, various adjuvants and/or antigens can be carried for prolonged release and protection, specific ligands can be integrated into particles to promote uptake by dendritic cells and macrophages inducing systemic and mucosal immunity [111] or co-stimulatory signals (CD-80, CD-44) and pro-inflammatory cytokines (IL-6/12) to enhance T-cell activation and expansion, and multiple immune signalling pathways can be synergistically modulated. The main nanoscale-based materials and platforms used in vaccines against animal pathogens are now discussed.

### 3.1. Protein Nanoparticles

There are many types of protein NPs; however, many intrinsic challenges are crucial for achieving ideal targeting and widespread inclusion in vaccine formulations. In spite of that, protein nanoparticle platforms improve the immune response to antigens in vaccines having tremendous potential for immunisation since proteins are antigenic and nanometer-sized (as are other biomolecules such as oligonucleotides and polysaccharides). Antigen presentation becomes increased when attached to a nanoparticle platform and T-cell epitope introduction enables inducing stronger immune responses. Antigen valency and spacing are essential for achieving such desirable host responses [112].

#### 3.1.1. Self-Assembling Proteins

Several highly oligomeric non-viral proteins (mainly the heat-shock protein, ferritin and E2) can self-assemble and assemble other proteins or biomolecules and thus present antigens on these NPs to immune system cells. Engineered assembly enables custom precision and diversity of structures and functionalities offering a number of advantages, including multivalency; immunisation against infectious diseases has thus been promoted [76]. Protein self-assembly enables atomic scale precision regarding the final architecture, having remarkable diversity of structures and functionalities, although defining the required self-assembling interactions, motifs, and immunological properties constitutes a critical challenge for the design of protein-based nanoparticles. The structure’s supramolecular symmetry (nanorings, polyhedral cages, nanotubes, nanofilaments, etc.) and ordered architecture appear pivotal. Moreover, protein nanoparticle type and specific functionalisation time with antigenic determinants is key to obtaining stable nanovaccines. Functionalisation could also be carried out with adjuvants, immuno-stimulating molecules, etc. on the same protein/peptide nanoparticles to modulate immune responses. Specifically, self-assembled peptides seem to offer important advantages and it has been envisaged that modern computational technologies may favour the macromolecular engineering of immunostimulatory peptide assemblies, leading to improved vaccine immunogenicity, efficacy, and safety [77,113,114].

#### 3.1.2. Virus-like Nanoparticles (VLPs)

Viral-like particles (VLPs) have virus-like nanostructures (sub-viral particles) lacking infectious genetic materials and exposing repetitive subunits on the particle surface, thus providing safety and inducing strong immune responses. So, VLPs are natural subunit nanoparticle platforms, although they may be produced via recombinant technologies retaining similar antigenic features of their viral capsids (viral vector backbone) with the potential of being polyvalent (have repetitive structures which can provide arrays of immunogenic antigens) and self-assembled. These engineered viral virus/particles, formed spontaneously by interactions between viral structural proteins, can mimic infection by entering host cells and inducing host–cell expression of foreign heterologous antigens using viral mechanisms. Hence, VLPs are highly immunogenic/antigenic and are able to elicit broad immune responses by different pathways to those used for conventional viral vaccines because they are processed in both MHC class I and II providing several benefits, including immunostimulatory activity. VLP formulation with other adjuvants (Chitosan, pattern recognition receptors agonist adjuvants, etc.), immune-modulators (cytokines such as IL-12) and excipients/preservatives may even optimise their properties, stimulating innate and adaptive immune responses [115,116]. All these make VLPs potential immunogens for veterinary vaccines. Other viral vectors such as the adeno-associated virus have also been successfully used as nanovaccine platforms [76,117]. VLPs can be produced in mammals, plants, insects, bacteria, cell cultures, and cell free systems, although involving some limits and inconveniences such as carrying host cell proteins and DNA. Their structure (having properties similar to those of extracellular vesicles) enables delivery of bio- and nanomaterials and is the basis for their classification (mainly enveloped/non-enveloped and natural/synthetic). Their tertiary structure is especially important as it simulates the original virus particle, thereby natively stimulating the immune system. All the above makes VLPs not just viable options to preventive and therapeutic vaccines but also as vaccines against different diseases and types of cancers or diagnostic antigens [118,119,120].

### 3.2. Lipid-Derived Nanoparticles (LNPs) and Nanomaterials: Liposomes and Virosomes

A liposome is a spherical self-assembled bilayer phospholipid (spherical soft NP) with particular structure, including a hydrophilic core. These LNPs have great flexibility and potential for chemical modification by conjugating hydrophilic and hydrophobic polymers, ligands, and molecules, such as DNA, RNA, and peptides. LNPs are formulated using precise molar ratios of phospholipids “helper lipids”, cationic-ionisable amino lipids, poly(ethylene) glycol (PEG)-lipids, and cholesterols. The intracellular protein expression of mRNA can be changed by modifying poly-(ethylene) glycol (PEG)-lipid molar ratios. This provides liposomes with advantages over many nanocarriers for use as vaccine adjuvant delivery systems [121,122]. NPs are especially advantageous for the nucleic acid delivery. Non-viral options such as liposomes using cationic lipids to facilitate the encapsulation of negatively charged nucleic acids such as mRNAs and neutral lipids to increase transfection efficiency have revolutionised vaccinology during the COVID-19 pandemic; they were previously used for preventing infectious diseases, treating cancers, and are now highly prized in oncology [123,124,125]. Many technological advances in the fields of structural vaccinology, synthetic biology, and adjuvants have made mRNA-LNPs formulations a promising nanovaccine platform for effectively controlling a variety of diseases, including infectious diseases [126]. Nucleic acid platforms are among the most promising in terms of safety, immunogenicity, efficacy, ease of manufacture, adaptability to various targets, and ease of biological delivery (due to cytoplasmic translation, avoiding nuclear targeting, and nuclear membrane passage difficulties). Nanovaccines mimic viral infection to express native antigens in the cytosol, inducing complete immune responses (in addition to their intrinsic adjuvant properties due to recognition by specific pattern recognition receptor). Quality attributes regarding mRNA constructs efficiently expressing a gene of interest have been identified, including 5′ capping efficiency and structure, UTR structure, length, and regulatory elements, encoding sequence modification, poly-A-tail properties, and mRNA purity [127]. Self-amplifying mRNA vaccines can be engineered by creating replicons having the replicative features of positive-sense, single-stranded RNA viruses, which promotes increased expression and immunogenicity, enabling lower dosages for an extended duration. Including multiple antigens in the same replicon makes this a versatile platform. Considerable infectious disease vaccinology-related preclinical research and clinical trials have led to the recent success of self-amplifying mRNA vaccines against COVID-19, mRNA technology, and LNP [107,128,129].

Lipid nanomaterials (virosomes) are fabricated by expanding phospholipids and biomaterials acting as nanocarriers and having promising effectiveness against viral infections. These spherical and unilamellar vesicles are enveloped with viral phospholipids having a removed nucleocapsid able to adsorb antigen epitopes and integrate them into a phospholipid bilayer. Their surface may be modified with polymers or moieties to target different types of host cell (acting as nanoscale-based adjuvants) and promote potent humoral and cellular immune responses having excellent tolerance and safety profile. The critical factor for achieving this seems to be epitope/fusion protein location in the virosome structure, being versatile vehicles for cargo delivery [130]. Another novel carrier has been suggested recently, based on vesicular systems consisting of non-ionic surfactants as an efficient bilayer instead of liposomes to construct virosomes (niosomal virosomes) [131].

### 3.3. Polymeric Nanoparticles (PNPs)

Natural (such as chitosan, gelatin, hyaluronic acid, and alginate) and biocompatible and biodegradable synthetic polymers including chitosan derivatives, polylactic acid (PLA), poly lactic-co-glycolic acid (PLGA), polyethyleneimines (PEI), polyamidoamine (PAMAM), and polyesters are multifunctional nanocarriers; they can interact with immunocompetent cells to induce long-lasting immune responses. These polymers can modulate immunostimulatory properties by selecting the nature of the polymer shell and antigen positioning on it; the complementary formulation of various polymers may further improve and combine each of the properties in the final nanomaterial [132,133,134]. PNPs have been included in nanovaccines against pathogens due to important advantages such as preventing antigen degradation and clearance, with enhanced uptake by professional antigen-presenting cells, leading to effectively enhancing immune responses [135,136,137]. They are also suitable for delivering nucleic acids (in spite of a number of challenges, mainly low transfection efficiency and potential cytotoxicity) and acting as adjuvants and carriers in mucosal vaccines, having great potential for practical applications [25,123,138].

### 3.4. Inorganic and Other Nanoparticles

Inorganic gold nanoparticles (AuNPs) are highly stable for DNA/mRNA vaccine delivery; biodegradable nanomaterials such as micelles or polyanhydride NPs have favourable properties such as immune regulation, biocompatibility, biodegradability, mucosal adhesion, non-immunogenicity, low toxicity, and safety [139]. Oil-in-water nanoemulsions have been the most successful influenza vaccine adjuvants in humans and swine, making these potential veterinary adjuvants. TiO_2_ nanoparticles are the most widely used inorganic nanoparticles and can offer a new platform for constructing stable and functional emulsions. Including essential oils seems to have a strong synergistic antiviral effect, and when included in nanoemulsions, act as an immunisation vehicle to deliver/present viral proteins to the host’s immune system. Optimising several key factors such as particle size and stability can maximise water concentration, oil phase, and modes of carrying. This system is an efficient vaccine adjuvant for enhancing the adaptive response and many nanoemulsions have been proven safe [140,141,142]. There are other promising nanostructures such as nanosponges (very small sized and having a porous structure) or extracellular vesicles which are well suited as new delivery and/or immunomodulatory systems. Other recently introduced advanced nanomaterials such as cylindrical single wall or multiple wall nanoparticles (named carbon nanotubes), mesoporous silica nanoparticles or nano-disk systems based on nanodisk particles consisting of a disk-shaped phospholipid bilayer have broad positive properties and applications for inclusion in nanovaccines due to their functionalisation capabilities and multiple functions [143].

## 4. Latest Nanovaccine Applications Regarding One Health Relevant Pathogens

**Figure 2:**
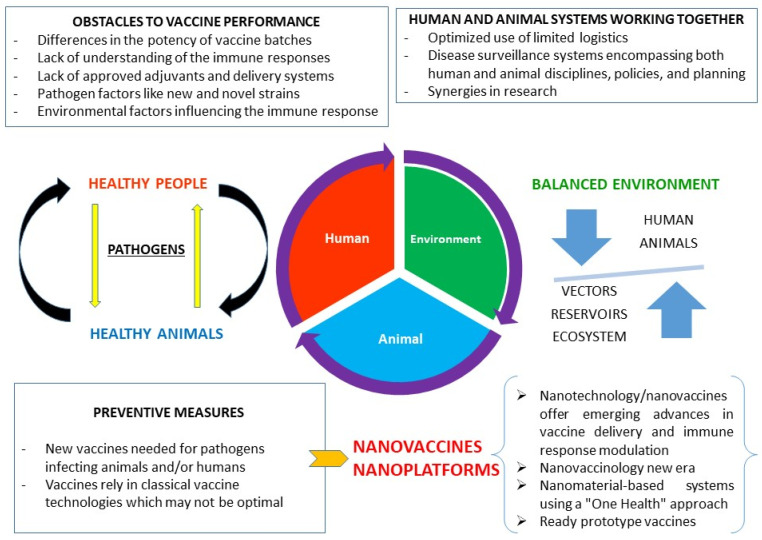
Nanovaccines as key elements regarding a One Health approach as preparation for future threats.

There are many types of nanoparticles but non-infectious viral particles (VLPs), self-assembling proteins, micelles, liposomes, inorganic particles, and polymers are the main systems increasingly used in nanovaccine preparation in the veterinary field. Third-generation vaccines are becoming one of the 21st century’s fundamental technologies given their ability to provide a rapid response, mainly regarding emerging infectious diseases impacting human and animal health [144]. Some data are now given concerning the latest advances in nanovaccines against animal pathogens, even though mention is made of important human diseases due to their relevance for nanovaccine development and progress, and regarding a One Health strategy for correct management (Figure 2).

### 4.1. Nanovaccines and Viral Infections

The main nanovaccine applications and advances have been made in the field of infectious diseases, more specifically against viruses. More than one hundred viral proteins from 35 virus families have been assembled into natural or synthetic VLPs or virosomes [118]. Many nucleic acid-based vaccine nano-strategies can be used to increase their immunogenicity and efficacy, such as cationic liposomes, polymer NPs (modifying them to respond to changes in pH to avoid degradation by lysosomes), inorganic gold/silver NPs, or carbon NPs. Several functional NPs (such as mesoporous silica or nano-disk systems) have been shown to have high antiviral activity against many viruses and can serve as a suitable vaccine delivery system as an alternative to conventional adjuvanted vaccines against animal viruses [113]. Numerous outbreaks of disease over the past 20 years (swine flu and swine pests, the Ebola epidemic in Africa, the Zika epidemic) and the recent COVID-19 pandemic have challenged us to prepare for the risks involved in the emergence/re-emergence of these and other unknown and highly contagious pathogens. The best way to confront this is with safe vaccines offering high and long-lasting protection. The WHO has called for intensified efforts against ten priority diseases: COVID-19, Crimean-fever, Ebola, Marburg disease, Lassa fever, MERS, severe acute respiratory syndrome (SARS), Nipah virus infection (NiV) and henipaviral diseases, Rift Valley fever, Zika, and disease X (WHO definition of a serious international pandemic that could be caused by a pathogen currently unknown to cause human disease). Major advances, using nanovaccines, have already been made against many of them and others of interest regarding animal health [145,146].

Self-assembling and metallic nanovaccines against the SARS-CoV-2 virus are the most promising for reducing the pathology. The mRNA-based ones are complex lipid nanoparticles formed by nanoprecipitation by mixing different lipids (at certain molar ratios) in ethanol with citrate buffer, salts to keep the pH close to body pH, and the mRNA encoding the viral S protein having a modified nucleotide sequence so that it actually encodes a trimer from its receptor binding domain (ACE2). This enables an ionisable lipid portion that can self-assemble, favour RNA encapsulation, and avoid endosomes and form stable particles in serum with low cytotoxicity. It also provides a phospholipid that stabilises lipid bilayer structure and envelops and reduces protein specific binding, thereby increasing half-life, escaping the barrier of the endothelial reticulum system and favouring their uptake by cells. This platform has thus enabled the development of highly effective vaccines and relatively easily and rapidly identifying and producing vaccine candidates against new viral strains. These characteristics and the fact that they are safe (as they are not infectious), cheap, and do not need to reach the cell nucleus have led to the first FDA-approved vaccine against COVID-19 being a nanovaccine.

Other protein and subunit-based platforms are also trying to develop nanovaccines, the so-called nanosponges in which nanoparticles are coated with membranes of lung epithelial cells or macrophages and which, after intra-tracheal administration, can neutralise infection and protect against other emerging coronaviruses [147]. Other platforms develop nanoparticles that are much better able to control the molecular conformation of the proteins that bind to them and vaccine outcome, using recombinant VLPs that carry antigens for all virus structural proteins or recombinant S protein gold nanoparticles (AuNPs) to enhance their immunogenicity. The aim is to achieve herd immunity that can lead to virus elimination, as has already occurred with two human and two other pathogenic avian influenza viruses after reaching this state. Ideally, this would be achieved by preventing virus entry through the respiratory tract and this is being pursued with experimental nanovaccines that can be administered by inhalation to induce mucosal immunity. Such vaccine mimics a virosome having virus-like double-stranded RNA that serves as an adjuvant to stimulate innate responses by stimulating TLRs, lung surfactant liposomes as the structure of the virus and the receptor-binding domain to fully simulate SARS-CoV-2 structure and completely block infection in mice for 5 months [148]. Work is underway to induce mucosal immunity against avian gammacoronaviruses causing infectious bronchitis using DNA nanovaccines encoding the virus’ nucleocapsid; these are nanotransported, together with several natural adjuvants such as chitosan and Quil-A that form nanoparticles. Upon administration they induce immune responses that reduce viral shedding in tear fluid and the trachea. Such data provide hope for protection against respiratory viruses in humans and animals. Novel approaches such as virosome-based vaccines against viral impacting animal diseases have also been developed and tested, i.e., Newcastle disease [149]. The authors reconstituted the viral membrane of a local field strain responsible for field outbreaks into a virosome; the virosome vaccine had good seroconversion and challenge with the virus in broiler chicken showing increased protection levels compared to commercially available vaccines.

Nano-vaccine platforms have been successfully used against Classical Swine Fever (CSF) when developing vaccines against difficult-to-control swine viruses. These are nanovaccines based on the virus’ E protein bound to ferritin (which has already shown promising results as the basis for a nanovaccine against FMD) or mi3 self-assembling proteins which induce strong humoral and cellular neutralising responses, even against different genotypes. This could be very useful in preventing future CSF panzootics [150]. Bivalent nanovaccines are being developed that confer protection against two swine viruses simultaneously; i.e., porcine circovirus 2, to which pigs are highly susceptible; and which prompts other co-infections such as influenza A virus. Chimeric VLP-based nanovaccines can self-assemble the circovirus matrix protein 2 exposed domain which induces cross-protection against other viruses. Although the nanovaccine has not been shown to protect pigs against circovirus challenge, it has been shown to induce high levels of specific NAbs in mice and pigs and differentiate vaccinated from unvaccinated animals, thereby increasing practical interest in this vaccine against circovirus. However, it confers protection against influenza A challenge and blocks influenza transmission. Thus, this type of bivalent nanovaccine has the potential to act as a universal vaccine against influenza A virus and block its reassortment and cross-species transmission [151,152]. Chimeric VLP-based nanovaccines against rabies and FMDV co-expressing a novel fusion rabies glycoprotein have been recently investigated. The authors also optimised VLP production in cell cultures by adding additives and demonstrated the production of specific Abs against both zoonotic diseases in mice, serving as a proof-of-concept recombinant FMDV vaccine candidate which would be easier to produce and safer than currently available vaccines [153]. The promise of VLPs is highly remarkable against fish pathogenic viruses in which delivery methods are inefficient. The oral route administration of several kinds of VLP preparations (including those expressed by cell-free expression systems) in experimental fish has resulted in equal responses to routine vaccine injection, making these suitable options for preventing many fish diseases [154,155]. Intraperitoneally administered VLPs produced in *Pichia pastoris* against betanodavirus (even without adjuvants) have shown dose-dependent, long-term protection against the disease in sea bass (never found before). The potential to simulate natural conditions during infection and pathogen challenge (not by injection) will elucidate whether these nanovaccines protect against infection [156].

Work has been carried out on the development of nanovaccines against some important arboviruses that have a great medical-veterinary impact, i.e., Dengue, for which the commercially available vaccine does not protect against Dengue virus serotype 4 (DENV-4). Many types of material have been used (mainly lipid nanoparticles) for subunit vaccines and RNA vaccines encoding DENV, ZIKV, and CHIKV structural proteins. Synthetic nanovaccines against Dengue flavivirus have recently been investigated by constructing four multi-epitope peptides linking structural protein epitopes for each of the serotypes with other linkers with epitopes that activate universal T-helper responses as well as CD8 T- and B-epitopes identified in silico, conserved for the four serotypes. This also increases the length of the peptide, making it more immunogenic. These multi-epitope peptides were conjugated to polystyrene particles to serve as a powerful adjuvant. The serotype 3 peptide construct was found to induce NAbs against several serotypes after the mice had been vaccinated, suggesting that this highly immunogenic nanovaccine could be improved into a tetravalent vaccine [157].

Chimeric peptides based on the CHIKV structural E2 glycoprotein functionalised with an amyloid protein have been tested regarding their ability to self-assemble and form fibril-like nanoparticles. Such nanoparticle arrangement gives them a superior ability to expose glycoprotein epitopes as well as being cytocompatible and efficiently taken up by macrophages. Thus, they do not require additional adjuvants to induce Th1 and Th2 responses having high specific IgG titres in mice [158]. Trials involving an mRNA nanovaccine encoding a monoclonal antibody (mAb) identified in patients who have survived natural CHIKV infections having high neutralising ability are also of interest. A nanovaccine administered in liposomes to mice has been shown to provide high protection against viral challenge; results from its administration in primates suggest that it may be used to prevent disease in humans [159].

Attempts have been made to develop nanovaccines against ZIKV, based on the virus’ non-structural proteins (infection, replication, and pathogenesis) having low immunogenicity; they were modified to self-assemble into nanoparticles, a novel technology used in cancer therapy but not validated for its ability to activate the immune system. The pilot strategy with this nanovaccine succeeded in making the NS1 protein highly immunogenic by inducing a high specific Ab response in mice, thereby demonstrating its potential usefulness for the development of novel subunit-based nanovaccines against ZIKV incorporating viral protein antigens interacting with host cell receptors [160]. However, studies of nanovaccines against arboviruses appear to require a good understanding of the types of immune responses induced, murine models of infection against many arboviruses (which do not exist in immuno-competent mice), and detailed studies of viral load and tissue damage in primates, all of which require large investments to determine such nanovaccines’ real potential [161].

### 4.2. Nanovaccines and Bacterial Infections

Nanoparticles facilitate the development of specific and long-lasting immunity in bacterial diseases at many levels. As they can also alter host microbiota composition, they can alter the synergy between host and microflora during immune responses and increase resistance to infection. Bacterial immunostimulators include secreting membrane vesicles, which are nanostructures having vaccine potential against bacterial infections. Such vesicles can serve as vaccines that include several antigens and stimulate T-cell responses against antibiotic-resistant bacteria such as *Staphylococcus aureus*. Thus, extracellular vesicles secreted by resistant strain surfaces coated with indocyanine-loaded magnetic silica nanoparticles (a promising material in cancer immunotherapy) could significantly reduce *S. aureus* infection (surface infection and systemic invasiveness) in experimental models and had therapeutic effects against resistant strains, which also appeared to prevent infection complications [162]. Avian pathogenic *Escherichia coli* outer membrane vesicles from the O2 serotype have been experimentally shown to promote both non-specific and specific protective immune responses against homologous infection in broilers by reducing bacterial loads and proinflammatory cytokine production and activating T-cell responses, mainly Th1 [163].

Carbon nanotubes and poly-anhydrous NPs have been tested in other murine experimental models, in both cases generating strong immune responses. The carbon nanotubes were able to absorb and present the *Anaplasma marginale* MSP1 protein on both their outer and inner surfaces and pass through biological membranes without affecting their integrity. The poly-anhydrides encapsulated whole cell lysates and *Mycobacterium paratuberculosis* culture filtrates by nanoprecipitation and proved to be very safe. Both nanovaccines provided more effective, homogeneous, and stable responses than commercial ruminant vaccines (peptide adjuvanted Anaplasmosis or inactivated Johne’s disease vaccines) opening up new vaccine perspectives for the control of intracellular pathogens [164,165]. Polyanhydride formulations including a lipopolysaccharide O-antigen-deficient rough *Brucella abortus* mutant from a virulent field strain have been tested for extended antigen release in cattle via implants to mimic the kinetics of antigen availability during persistent infection. The results suggested sustained cellular responses against *Brucella* antigens without developing tolerance. Moreover, nanovaccines based on the oligopolysaccharide antigen and PLGANPs have shown promise against *Brucella melitensis* via efficient opsonophagocytosis [166,167].

Poly-anhydrous nanovaccines and chitosan nanovaccines are safe and effective against *Salmonella* orally. However, their direct administration into eggs may be much more efficient in inducing specific systemic and mucosal responses, thus reducing caecal colonisation by *Salmonella enteritidis* in broilers, as has been demonstrated with chitosan NPs that carry the bacterium’s outer membrane protein on their surface together with bacterial flagellin extracts [168]. Combining two nanoadjuvants can enhance protection against bacterial challenge, i.e., using a single-dose combination nanovaccine comprising polyanhydride nanoparticles encapsulating pathogenic fusion proteins co-adjuvanted with noncanonical CDG adjuvants. These are the latest innovations against highly virulent *Yersinia pestis* and *Bacillus anthracis* in which the combinatorial experimental nanovaccine rapidly induced specific protective Ab responses mediated by lower levels of NO and ROS even with toxin neutralisation ability but also long-lived Ab responses, very relevant for bioterrorism agents such as these [169,170]. Another highly contagious bacterial fish disease (columnaris) is caused by *Flavobacterium columnare* having characteristic lesions in the mucosa of tilapia. Farmed stocks could benefit from an experimental mucoadhesive polymer chitosan-complexed nanovaccine applied by immersion, having up to 89% protection levels. It seems that positively charged vaccine nanoparticles comprising a bacterial formalin-killed isolate in a nano-emulsion with chitosan mimic live bacteria’s physical and biological characteristics and promote stronger binding to negatively charged mucosal membranes [171]. Chitosan-based nanovaccines have been recently tested against avian pathogenic *Escherichia coli* highly impacting poultry production worldwide. Through loading or encapsulating the outer membrane protein and flagellar antigen (O-F antigen), improved vaccine efficacy and protection was observed in chickens compared to basic or Montanide ISA 71 R VG adjuvanted against *E. coli* O1 and O78. These two main serogroups are frequently associated with disease formation in poultry farms, pointing out the potential of biomimetic nanoparticles for developing universal anti-diverse serotype vaccines [74,172]. Biocompatible chitosan nanovaccines containing *S. enteritidis* O-F antigen and a surface coated with F-protein (acting as a potent mucosal adjuvant) are effective orally, boosting specific memory T- and B-cell responses. The nanovaccine targets and activates immune cells in the mucosa, providing evidence of robust mucosal secretory IgA Abs and cell-mediated immune responses reducing the challenge of *S. enteritidis* load in chicken intestines and against other important bacterial and viral infections in chicken and pigs [173,174,175].

### 4.3. Nanovaccines and Parasitic Infections and Infestations

Vaccine development (including nanovaccines) and approval in the field of parasitology lags significantly behind that for viruses and bacteria; protozoan diseases such as malaria have been known for 2500 years and, although introductory pilot trials have been carried out for RTS, S (or Mosquirix, a vaccine partially protective in children), no vaccines have been licensed for widespread use, despite efforts during the last 35 years [176]. The fact that protozoan parasites have a huge number of proteins compared to viruses makes it very difficult to identify the right targets and their evolved sophisticated immune-protective mechanisms highlight a number of challenges to be addressed. It has been known for over 50 years that CD8^+^ T-cell responses are essential for achieving immunity and nanovaccines are now being suggested as platforms for developing an effective vaccine [177]. However, efforts to develop malaria vaccines with nanomaterials date back to the last 5–6 years, in which antigens that block transmission to mosquitoes have been tested on the surface of gold nanoparticles and also on chimeric VLPs. These induced high titres of specific transmission-blocking Abs have similar functions to those of protective mAbs in mice [178,179,180]. Induced Ab ability to compete with protective experimental mAbs puts nanovaccine platforms in a very advantageous position for future malaria vaccine development. Virosomes represent other nanomaterial used recently against malarial parasites. Influenza virosomes have been adapted to a non-adjuvanted virosome-based PfCyRPA vaccine having a blood-stage *Plasmodium falciparum* cysteine-rich protective antigen on their surface. This formulation seems to be a suitable antigen delivery system inducing strong growth-inhibitory Abs in vivo and in vitro. The system and the candidate antigen can be further adapted as a multivalent and multi-stage virosomal anti-malarial vaccine [181].

Nanovaccines against *Trypanosoma cruzi* have been developed that activate polyfunctional T responses by subcloning G2 and G4 antigens into nanoplasmids and optimising their transport and expression. Their inoculation in experimental animals induces high control of parasite dissemination and replication (unlike traditional vaccines) and total protection against successive infections by the same isolate, which can help to efficiently manage Chagas disease and avoid anti-parasitic treatments having adverse effects and resistance [182]. Nano-optimisation of conventional options by encapsulating total antigen, subunit extracts, and recombinant proteins in biocompatible nanopolymers such as PLGA (having potent adjuvant properties able to induce higher immune responses) has achieved protection levels of up to 80% regarding leishmaniosis and the nematode *Haemonchus contortus*, and a significant reduction in the impact of parasites on organs in visceral Leishmaniasis and around 50% against the nematode in goats [183,184]. Major advances have been made in the development of nanovaccines against Toxoplasmosis using chimeric polypeptides based on flagellin protein and innate response stimulators together with major HLA allele epitopes, reducing *T. gondii* parasite load by up to 87% [185]. Very promising results have been achieved in laboratory models using antigen-absorbing silica vesicles as new nanovaccine platforms against hard ticks and tick-borne diseases [186]. It appears that combining two nanoplatforms (VLPs and spherical hollow silica vesicles as separate formulations) has synergistically enhanced efficacy for producing balanced immune responses against *Theileria parva*, especially sporozoite-neutralising activity, highlighting novel nanovaccination strategies [184].

## 5. Conclusions and Future Perspectives

Innovative successful nanovaccines against the SARS-CoV-2 virus have resulted from years of major development in vaccine technologies, research towards understanding the immune system, and host–pathogen interactions and reprogramming. Increased intensive farming has facilitated pathogen adaptation to new hosts, thus being closely associated with increased outbreaks of a variety of animal and zoonotic diseases [187]. Producing vaccines against a range of endemics and emerging or re-emerging pathogens is thus an urgent need because zoonotic infections jumping from animals to humans are increasingly realistic. The COVID-19 pandemic has definitely highlighted the potential advantages of nucleic acid and nanocarriers as vaccine formulations. Thus, mRNA-based nanovaccine platforms are best suited to serve as the basis to formulate innovative approaches for veterinary purposes and transformation if improvements can lead to single shot or needle-free cross-protective vaccines against several pathogens. These platforms provide an opportunity to integrate tailor-made adjuvant formulations containing multiple ligands involved in the activation of the innate immune response, providing appropriate immune responses.

Targeting the pathogen–immune system interacting cells is a must to orchestrate a desired immune response since disease phenotypes are related to the degree of the immune system’s dysregulation in response to pathogen infection. Vaccine immunomodulation may promote altered coordinated immune cellular interplay influencing dynamics regarding subsequent responses to other pathogens due to immune system plasticity. In-depth molecular knowledge of heterologous immunity during infection/disease phases and their inclusion in essential vaccinology research and subsequent clinical trials appear to be very important for optimising broader immune responses. Understanding the T-cell immunological response role and characteristics is essential, and pathogen-specific T-cell immune profile research may decisively help to identify key immune signatures and insights, benefitting the development of vaccines against impacting diseases [174,175,176]. Key specific immune signalling mechanisms and protection correlates must be identified for immune cascade reprogramming and designing innovative vaccines having predicted outcomes that can achieve superior and complete protection. Immune enhancement exacerbating the immune responses may complicate vaccine development; accordingly, a balanced induction of NAbs, antibody effector functions, and T-cell immunity against multiple pathogens’ antigens seems to be the aim to follow. Impressive advances with novel versatile platforms based on chimeric nanoparticles and versatile RNA chaperone functions have demonstrated that cross-protection against pre-pandemic viral diseases is possible using next-generation nanovaccines [177,178].

Nanovaccine development potential and its rapid scaling-up is facilitated as only a protein’s genetic code is needed and the mRNA can be produced synthetically, thus adding an important manufacturing advantage. Vaccinology technologies must move to global public health-related applications, mainly the “One Health” approach combined with modern omics (including glycomics and metabolomics), computational in silico strategies, protein modelling, genome editing engineering, and nanoparticles and nanocarriers’ great potential is essential for combating hyper-variable-impacting pathogens [27,188,189]. A roadmap of global interdisciplinary collaborations, focused coordinated programmes, and critical investment is thus needed to accelerate vaccine design and timely modern worldwide vaccinology developments and solutions, because the future of vaccines could depend on using different nanoplatforms to create prototype vaccines ready to reduce or block transmission and prevent future threats.

## Figures and Tables

**Table 1:** The main challenges of many commercial/in development vaccines for their practical application.

Vaccine Type	Benefits/Advantages	Constrains/Inadequacies/Shortcomings
Inactivated/live-attenuated vaccines	- Live-attenuated vaccines do not require adjuvant co-administration - Organisms lose their replication ability regarding inactivated or “killed” pathogen vaccines (e.g., by radiation, chemical or heat treatment) - Are multivalent (as they are made up by the whole pathogen, it is expected that they include more epitopes than single-antigen subunit vaccines) - Potent response	- Short-term protection (mainly in inactivated vaccines) - Provide a brief period of protection - Lack of/limitations regarding effective adjuvants - Inadequate administration route (i.e., for pathogens invading mucosal surfaces or when in ovo vaccination is required) - Public safety concerns/ethical limitations - Limited efficacy (inability to induce cell-mediated immune responses) and duration of immunity - Risk of reversion to virulence in vivo - Can be lethal and dangers are detected a posteriori - Inability to differentiate naturally infected from vaccinated animals (DIVA) due to traces of pathogen non-structural proteins - Unable to eliminate pathogens from carrier animals - Potential for undetected, subclinical infections in vaccinated animals - Viral shedding and subsequent recombination between field and vaccine strains (although transmission may be highly desirable for wildlife vaccination) - Not amenable to genetic changes - Cultivation of virulent pathogens and risk of escape from production sites - Environmental and associated regulatory concerns - Other risk factors such as allergies or off-target responses
Subunit or recombinant vaccines	- Mostly consist of protein immunogens or antigens - Favourable response - Induce an innate immune response (RNA vaccines) - Very good safety - Rapid and scalable production (recombinant and RNA vaccines) - Unable to cause the disease - Ability to differentiate naturally infected from vaccinated animals	- Constraints on efficacy, application, and production - Unstable efficacy - Poorly immunogenic: require strong adjuvants - Low levels of specific protective neutralising antibodies (incomplete protection) - Expression systems are often not feasible (lack of post-translational modifications and folding requirements)
All	- Beneficial effects for society in terms of reducing disease transmission and outbreaks	- Limited success - Vaccines may not protect from becoming infected - Stability concerns: a cold chain needs to be maintained to preserve vaccine efficacy - Vaccine immunogenicity - Lack of knowledge regarding the major virulence factors - Low level of cross-protection/lack of multivalent vaccines (some only protect against homologous serogroups, do not contain all the virulent circulating serovars involved in an outbreak) - Lack of suitability for oral applications in mass-scale wildlife vaccination - Potentially severe side-effects - Protective immunity may cause immune-mediated diseases - Some lack the ability to protect against infections’ symptomatic form - Some fail to induce protective immunological memory - Efficacy against new and emerging strains varies - Some are less amenable to fast vaccine production

**Table 2:** Some ultimate effective options enhancing nanoscale adjuvanticity and delivery.

Nanovaccine Complex	Compound/Adjuvant Properties	References
Self-adjuvanting moieties	Poly and polyhydrophobic amino acids acting as self-adjuvants inducing specific antibodies able to clear bacterial load	[96,97]
	Trimethyl chitosan alone or self-assembled with poly (anionic amino acid) can stimulate the highest levels of serum protective antibodies and nanovaccines’ opsonin-mediated killing potential.	[98,99]
	Biomimetic nanoparticles self-assembled with Toll-like receptor phospholipids and nucleotides agonist activating strong immune responses and serving as a safe, simple, and efficient approach for anti-tumour immunotherapy	[100]
Biodegradable polymeric nanoparticles	PLGA, PLA-PEG a copolymer of polylactic acid (PLA), polyethylene glycol (PEG) and their adjuvanted derivatives facilitate their release upon degradation of the matrix, having prolonged biodegradation properties	[101]
	A ROS-triggered nanoparticle-based antigen delivery system consisting of three-armed PLGA, conjugated to PEG via the peroxalate ester bond (3s-PLGA-PO-PEG) and PEI as a cationic adjuvant (PPO NPs)	[102]
	Novel chitosan derivatives (aminated chitosan and aminated plus thiolated chitosan) promote strong mucoadhesiveness and thereby systemic and local immune responses following nasal vaccination	[103]
Liposomes-mRNAs	Incorporating lipid moieties into peptide epitopes increases antigen immunogenicity	[104,105]
	IgM (after spontaneous absorption on the nanosurface) serve as self-adjuvant by regulating antigen-presenting cell recognition and complement activation	[106]
	Carriers consisting of non-encoding RNA complexed with protamine (a cationic protein activating TLR7) naked 1-methylpseudouridine modified mRNA to small-molecule TLR2 and TLR7 agonists	[107]
Lipid-PLGA nanoparticles	Hyaluronic acid (HA)-decorated cationic lipid-poly(lactide-co-glycolide) acid (PLGA) hybrid nanoparticles (HA-DOTAP-PLGA NPs)	[108]
Nanoemulsions	A self-assembled biocompatible cationic-covered with hyper-branched poly(ethyleneimine) nanoemulsion has superior adjuvant activity than the non-cationic and traditional adjuvants in vivo.	[109]
